# Triple-targeting nanosystems with synergistic effects on iron Trojan horse, fluoroquinolone antibiotics, and photodynamic therapy specifically kill intracellular MRSA

**DOI:** 10.7150/thno.109374

**Published:** 2025-08-16

**Authors:** Kuiyu Meng, Liwen Yuan, Lulu Feng, Yaoyao Zhang, Hao Wu, Jie Zhang, Mubbashar Abbas, Wei Qu, Dongmei Chen, Shuyu Xie

**Affiliations:** 1 National Key Laboratory of Agricultural Microbiology, Huazhong Agricultural University, Wuhan, Hubei 430070, China.; 2 Hubei hongshan Laboratory, Wuhan, Hubei 430070, China.; 3 National Reference Laboratory of Veterinary Drug Residues (HZAU), Wuhan, Hubei 430070, China.; 4 Key Laboratory of Prevention & Control for African Swine Fever and Other Major Pig Diseases, Ministry of Agriculture and Rural Affairs,Wuhan, Hubei 430070, China.

**Keywords:** Iron metabolism, Methicillin-resistant *Staphylococcus aureu*, antibiotic resistance, therapeutic agent

## Abstract

**Rationale:** Iron is necessary for the survival of microorganisms. The uptake network is highly expressed by host cells and methicillin-resistant *Staphylococcus aureus* (MRSA) and competes with heme iron. Gallium porphyrin (GaPR), a heme mimetic compound, was synthesized to develop an innovative nanosystem as a triple-targeting agent for uptake network recognition. GaPR is also used as a dual therapeutic molecule of "iron trojan horse" and photosensitizer to achieve synergistic antibacterial effects with levofloxacin to eradicate intracellular MRSA—a problem that conventional therapeutic techniques cannot overcome due to limited drug penetration, antibiotic resistance, and off-target effects.

**Methods:** A library of hemimetic compounds was synthesized. GaPR was selected as the optimal candidate owing to its antibacterial activities and competitive binding affinity for iron uptake receptors. The optimal GaPR and the photosensitizer tetrakis-(4-carboxyphenyl)-porphyrin (TCPP) were used to prepare a levofloxacin (Lev)-loaded zirconium-based organometallic scaffold (Lev-GaPR-PCN). Hyaluronic acid (HA) was linked to the Lev-GaPR-PCN surface via ROS-reactive thioketal bonds (TK). The triple-targeting performance and synergistic efficacy of HA-Lev-GaPR-PCN against intracellular MRSA were tested *in vitro* and *in vivo*.

**Results:** GaPR showed strong bactericidal activity against MRSA by interfering with iron metabolism. GaPR-PCN exhibited excellent binding ability with host-derived heme-binding proteins (Hpx/LRP1 and Hpg/CD163) and the iron-regulated surface determinant (Isd) system of MRSA for infection site, infected cell, and intracellular targeting. HA coating enabled covert circulation and decreased nonspecific uptake by healthy cells (< 5% fluorescence intensity after 6 h) while promoting infection-induced release via hyaluronidase and ROS. *In vitro*, HA-Lev-GaPR-PCN achieved 3.42-fold greater colocalization with intracellular MRSA (Pearson correlation: 0.41 vs. 0.12 for PCN-224 controls) and decreased the extracellular/intracellular minimum inhibitory concentrations (MICs) of Lev under PDT from 8/64 μg/mL to 1/2 μg/mL. *In vivo*, it resulted in prolonged retention (72 h vs. 36 h) and a 1.5-2.5-fold greater fluorescence intensity at infection sites for non-HA nanosystems. Compared to Lev alone, it decreased the bacterial load by 501-fold (2.7 log) and abscesses (diameter: 0.6 cm vs. 3.3 cm) by combining chemical, metabolic, and physical antibacterial mechanisms without causing toxic effects.

**Conclusion:** This study represents a paradigm shift in intracellular infection therapy for MRSA and other resistant bacteria using a hemimetic compound as a triple-targeting and dual therapeutic agent that provides a streamlined, clinically feasible solution with high efficacy and specificity.

## Introduction

Methicillin-resistant *Staphylococcus aureus* (MRSA) is a gram-positive pathogen that persists intracellularly; it is one of the most common causes of hospital-acquired infections worldwide 1-3. Although many antimicrobial agents have been developed, MRSA bacteremia has very high mortality rates of 15-50% 4. A key therapeutic challenge arises from the ability of MRSA to evade immune defenses by colonizing host cells, which leads to chronic or recurrent infections. This intracellular niche not only protects MRSA from host defenses but also creates physical and pharmacological barriers that limit the efficacy of antibiotics. Effective treatment requires triple precision, involving targeted administration of agents to the infected site and host cells, followed by colocalization with intracellular MRSA at bactericidal concentrations.

Most conventional antibiotics, except for fluoroquinolones such as levofloxacin (Lev), show low clinical efficacy against intracellular MRSA due to their nonspecific biodistribution, inefficient cellular uptake, lysosomal degradation, and insufficient intracellular colocalization with bacteria. These limitations result in significantly lower intracellular drug activity, with an increase in the minimum inhibitory concentrations (MICs) against intracellular *S. aureus* by 16-32-fold compared to the MICs in planktonic cultures 7,8. Moreover, certain antibiotics accumulate less efficiently in infected cells than in healthy tissue. For example, Lev concentrations in MRSA-infected THP-1 macrophages are 50% lower than those in noninfected cells 7. These subtherapeutic intracellular antibiotic concentrations create selection pressure that drives the development of resistance and further exacerbates the clinical burden of MRSA infections, which are associated with higher mortality rates and healthcare costs than those associated with drug-susceptible strains 9. Vancomycin is the drug of first choice for treating MRSA, but the emerging resistance associated with the increasing clinical use of vancomycin threatens its long-term utility 10. This crisis highlights the urgent need for innovative therapeutic strategies that circumvent existing resistance mechanisms through nontraditional antibacterial modalities.

New findings emphasize the key role of competition between hosts and pathogens for essential nutrients, such as iron, in controlling bacterial pathogenesis. This interaction has led to new therapeutic strategies using metal-derived antimicrobial agents that depend on nutritional immunity, a concept that differs from that of conventional antibiotics. For example, siderophore-mimetic antibiotics use bacterial metal uptake systems to increase drug delivery, whereas metalloanti-microbial agents act as competitive antagonists of microbial nutrient receptors to disrupt survival pathways 11. Gallium-based agents have attracted considerable attention because of their unique iron-mimetic properties. Gallium is a nonfunctional iron analog that is taken up by bacteria via iron acquisition pathways (e.g., siderophores or heme transporters) and exerts antibacterial effects by interfering with iron-dependent processes such as DNA synthesis and redox homeostasis 12. Iron depletion is at the center of these processes and effects. During an infection, the host boosts immunity through nutrition to deprive pathogens such as MRSA of iron. In response, MRSA adopts virulence strategies, such as toxin-mediated erythrocyte lysis, to siphon off heme, the major source of iron in mammalian hosts. The iron-regulated surface determinant (Isd) system of MRSA allows direct heme extraction from hemoglobin. To disrupt the function of this system, gallium porphyrin (GaPR), a heme-mimetic compound in which iron is replaced by antibacterial gallium, was developed. GaPR functions like a "Trojan horse" and uses the heme uptake machinery of MRSA to enter bacterial cells. After it is internalized, gallium irreversibly binds to iron-dependent enzymes (e.g., catalases and cytochromes) and paralyzes metabolic and detoxification pathways 13. However, the systemic application of GaPR is associated with several problems. Nonspecific distribution and inadvertent uptake by host cells raise concerns about off-target toxicity, which limits therapeutic benefit 13. These limitations emphasize that targeted delivery systems need to be developed to improve precision and safety.

Along with metalloantimicrobials, photo-dynamic therapy (PDT) is a promising antimicrobial strategy. It uses light-activated generation of reactive oxygen species (ROS) to induce bacterial apoptosis or necrosis while minimizing the development of resistance, a key advantage over conventional antibiotics 14-16. Nanoscale metal-organic frameworks (MOFs) with porous crystalline structures of metal nodes and photosensitizers based on organic compounds are suitable for PDT. Their design improves light absorption efficiency and facilitates the transmembrane delivery of photosensitizers, eliminating the main limitations of free molecular drugs 17. For example, in another study, we showed that porphyrin-based MOFs effectively penetrate biofilms and kill metabolically active and dormant MRSA without inducing resistance 18. Despite these advancements, PDT and other innovative strategies, such as gallium-based antimicrobial metal agents, share a common challenge: targeting intracellular MRSA with conventional antibiotics.

Nanosystems offer potential solutions by exploiting infection-specific microenvironments (e.g., bacterial metabolic enzymes and increased redox stress) for site-specific activation or release 19,20. Functionalization with ligands that recognize host or pathogen receptors (e.g., transferrin receptors on infected macrophages and Isd proteins on MRSA) can further improve targeting accuracy 21-24. However, such strategies often require complex development and multistep functionalization, which makes clinical application considerably more difficult. The intracellular efficacy depends not only on the efficiency of cellular uptake but also on the subcellular transport and stability of the payload. Most nanosystems are transported via endosomal/lysosomal pathways, leading to premature degradation of the carriers and the encapsulated antibiotics before they reach intracellular MRSA. This emphasizes the need for rationally designed nanosystems that provide a triple point of attack: accumulation in infected tissue, selective penetration into infected host cells, and escape from lysosomal compartments to colocalize with intracellular bacteria. The combination of such a target with synergistic antibacterial mechanisms (e.g., ROS generation, iron interference, and antibiotic release) can overcome persistent intracellular MRSA infections and simultaneously inhibit the development of resistance.

**Figure 1:**
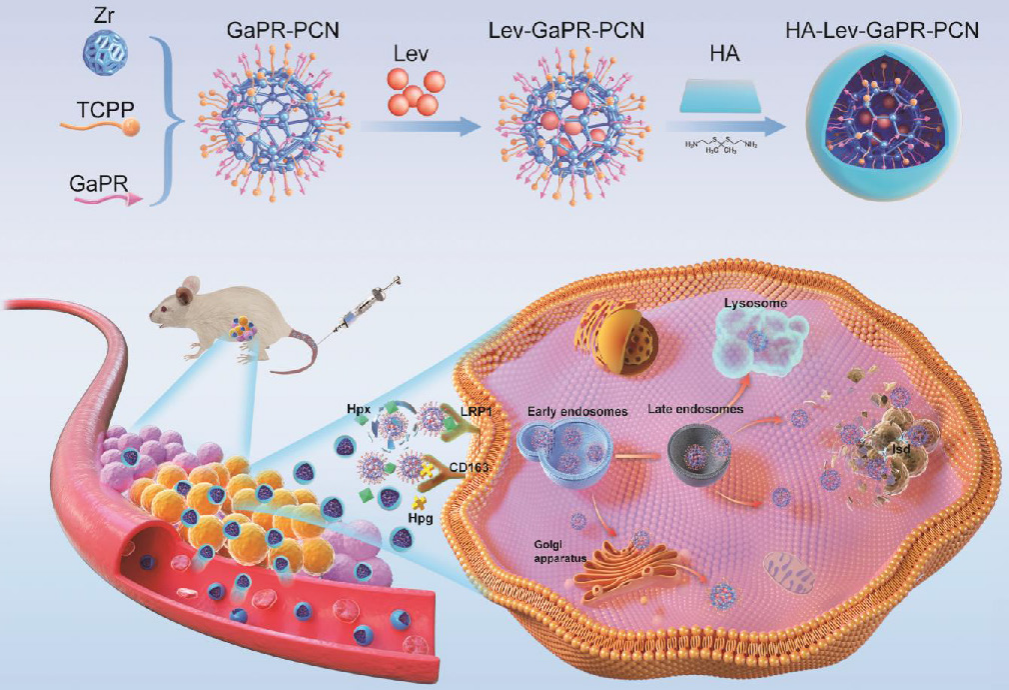
Design pattern of HA-Lev-GaPR-PCN and its programmed triple targeting to infected sites, infected cells, and intracellular MRSA via the over-expressed heme acquisition system of infected cells and MRSA.

Some studies have shown that host cells counteract intracellular bacterial infections by upregulating nutrient sequestration systems, a defense mechanism that limits the pathogen from accessing essential metals such as iron 25-32. Iron is a key cofactor in heme and is essential for host and microbial survival 26. During MRSA infection, host cells secrete hemopexin (Hpx) and haptoglobin (Hpg), which bind extracellular heme/iron and form complexes. These complexes are internalized via the overexpressed heme scavenging receptors of the density lipoprotein receptor-related protein (LRP1) and CD163, thus depriving MRSA of iron—a strategy termed "nutritional immunity" 27-32. Intracellular MRSA compensates for this by activating its Isd 33,34. Based on this dual adaptation involving host-induced iron limitation and pathogen-induced heme piracy, we developed a novel triple-targeting strategy that uses the common mechanisms of heme acquisition to achieve precise delivery and synergistic therapy. To exploit the common heme acquisition mechanisms of infected cells and MRSA, we designed, synthesized, and screened a library of heme-mimetic compounds to identify candidates with dual functionalities that are triple-targeting and antibacterial. Among these, GaPR, in which iron is replaced by antibacterial gallium, is an optimal agent owing to its structural mimicry of heme, its competitive disruption of iron metabolism, and its high affinity for the host (CD163/LRP1) and bacterial Isd receptors. GaPR and the photosensitizer tetrakis (4-carboxyphenyl)porphyrin (TCPP) were integrated as organic linkers into a zirconium-based porphyrinic MOF (named GaPR-PCN) via solvothermal synthesis. Lev was encapsulated into the mesopores of GaPR-PCN by electrostatic adsorption, resulting in a synergistic antibacterial effect of DNA gyrase inhibition of Lev, iron metabolism blockade of GaPR, and photodynamic ROS generation of TCPP. GaPR-PCN has potent antibacterial activity, but its systemic application is limited by Hpx-mediated scavenging activity in the bloodstream. To solve this problem, hydrophilic hyaluronic acid (HA) was grafted onto GaPR-PCN via ROS-cleavable thioketal linkers (TK), resulting in HA-Lev-GaPR-PCN. This design prolongs circulation by conferring "stealth" properties, preventing the premature release of Lev, reducing endocytosis by normal cells, and allowing infection-specific activation. At the site of infection, HA-Lev-GaPR-PCN undergoes a programmed activation cascade through which ROS (from inflamed tissue or laser-activated TCPP) and hyaluronidase (HAdase) degrade it, exposing the core of Lev-GaPR-PCN. The surface-exposed GaPR of Lev-GaPR-PCN selectively binds to overexpressed CD163 and LRP1 receptors on MRSA-infected cells after forming complexes with infection site-enriched secreted Hpg and Hpx, allowing efficient entry into infected cells. The nanosystem bypasses lysosomal degradation and anchors to the Isd system of MRSA via hemimetic recognition, ensuring that Lev, GaPR, and TCPP are delivered to intracellular MRSA undamaged. Upon laser irradiation at 660 nm, GaPR and TCPP generate ROS that synergize with Lev and GaPR to efficiently eradicate MRSA while preventing the development of resistance. This unique design ensures that HA-Lev-GaPR-PCN exhibits programmable triple-targeting and synergistic antibacterial effects without cross-resistance (Figure 1), representing a paradigm shift in combating intracellular MRSA through bioinspired and multistep targeting.

## Materials and Methods

### Materials

TCPP (purity: > 97%) was purchased from Ark Pharm (Chicago, USA). ZrOCl_2_·8H_2_O, methyl-β-cyclodextrin and chlorpromazine hydrochloride were obtained from Sigma-Aldrich (St. Louis, MO, USA). PR was purchased from Yuanye Co, Ltd. (Shanghai, China). HA (40-100kda, purity: > 99%) and amiloride hydrochloride were bought from Shanghai McLean Biochemical Technology Co., Ltd. (Shanghai, China). Lysozyme, RNeasy minikit Qiagen and ImProm II cDNA Synthesis Kit, ImProm II cDNA Synthesis Kit (Promega), and ChamO Universal SYBR gPCR MasterMix were obtained from Vazyme Biotech Co., Ltd. (Nanjing, China). Lev was acquired from Hubei Weideli Chemical Technology Co., Ltd. (Wuhan, China). N-(3-dimethylaminopropyl)-N-ethylcarbodiimide hydrochloride and 1-hydroxybenzotriazole were purchased from Shanghai Aladdin Biochemical Technology Co., Ltd. (Shanghai, China). Cytochalasin D was purchased from Bailingway Technology Co., Ltd. (Beijing, China). Hpx (HY-P70884) and Hpg (HY-P73934) were purchased from MedChemExpress (Shanghai, China). Anti-LRP1 (ET1601-1) and Anti-CD163 (ER1804-03) were obtained from Huaan Biotechnology Co., Ltd. (Hangzhou, China). Anti-*S.aureus* (ab20920) was purchased from Abcam Biotechnology Co., Ltd. (Wuhan, China).

### Cell lines

RAW264.7 cells were provided by the National Reference Laboratory for Veterinary Drug Residues (HZAU) (Wuhan, China). The bovine mammary epithelial cell line (MAC-T cells) was obtained from Procell Life Science&Technology Co, Ltd (Wuhan, China). The cells were cultured in high glucose DMEM supplemented with 10 % (v/v) FBS and 1 % antibiotics (penicillin/streptomycin, 100 U/mL) at 37 °C and 5 % CO_2_.

### Animal experiments

All animal experiments were conducted in accordance with the approval of the Animal Care and Use Committee of Huazhong Agricultural University (Wuhan, China) (approval number: HZAUMO-2021-0186, HZAUMO-2024-0208). 20 healthy 6-8-week-old female Balb/c Nude mice (SPF, 20±2 g) and 90 healthy 6-8-week-old female Balb/c mice (SPF, 20±2 g) were provided by the Laboratory Animal Centre of Huazhong Agricultural University (HAZU) (Wuhan, China).

### Preparation of GaPR-PCN

In a 250 mL round bottom flask, 300 mg ZrOCl_2_-8H_2_O, 100 mg TCPP, 100 mg GaPR and 2.8 g benzoic acid were added individually. Subsequently, 100 mL of N,N-dimethylformamide (DMF) was added to the flask. The mixture was stirred at 300 rpm for 5 h and heated to 90 °C. Subsequently, the resulting product was separated by centrifugation and washed three times successively with fresh DMF and acetone. On the other hand, PCN-224 was prepared using the same process, but without the addition of GaPR.

### Preparation of Lev-GaPR-PCN

A total of 40 mg GaPR-PCN and 10 mg Lev were dispersed separately and dissolved in 10 mL DMF. The mixture was then stirred at 300 rpm for 24 h at 30 °C to obtain Lev-GaPR-PCN. Lev-GaPR-PCN was obtained by centrifugation and dried in vacuo after washing three times. Similarly, 40 mg PCN-224 and 10 mg Lev were dispersed and dissolved in 10 ml DMF to prepare Lev-PCN-224 under the same conditions. The Lev concentration in the supernatant after centrifugation was determined by ultraviolet spectrophotometry. The drug loading capacity (%) = [(mass of added Lev - Lev in supernatant) / mass of dried Lev-GaPR-PCN] × 100.

### Preparation of HA-Lev-GaPR-PCN

To prepare HA-Lev-GaPR-PCN, 40 mg Lev-GaPR-PCN was dispersed in 40 mL deionized water at pH 7 containing 31.0 mg EDC and 11.5 mg NHS. The mixture was stirred at 300 rpm for 4 h, then 40 mg HA-TK-NH_2_ was added and stirring was continued for 24 h. HA-Lev-GaPR-PCN was obtained by centrifugation at 15 000 rpm, then washed three times with deionized water (pH=7) and dried under vacuum conditions. The same process was used for the preparation of sulphur-labelled HA-Lev-GaPR-PCN, except that sulfurized HA was used instead of HA.

### Degradation of HA shell of HA-Lev-GaPR-PCN

A concentration of 10 μg/mL HA-Lev-GaPR-PCN was incubated with both PBS solutions of pH 5.5 and 7.4 containing HAase at concentrations of 400 U/mL and culture medium containing 106 MRSA-infected RAW 264.7 cells (10^5^ cells/mL) for a period of 1 to 12 h.

At specific time intervals, the zeta potential and surface morphology of the incubated HA-Lev-GaPR-PCN were examined using the Zetasizer Nano ZS ZEN3600 analyzer and SEM (TESCAN MIRA LMS, Czech Republic), respectively. The degradation of the HA shell of the nanosystems was observed using a TEM with energy dispersive X-ray spectroscopy.

### Cellular uptake of Lev-PCN-224, Lev-GaPR-PCN and HA-Lev-GaPR-PCN

Cellular uptake of Lev-PCN-224, Lev-GaPR-PCN and HA-Lev-GaPR-PCN in RAW 264.7 and MAC-T cells was analyzed using a BD FACS Verse flow cytometer (Beckman Coulter, USA). Cells were seeded at a density of 2×10^5^ cells/well in 24-well plates. After 24 h of incubation, cells were treated separately with 10 μg/mL Lev-PCN-224, Lev-GaPR-PCN and HA-Lev-GaPR-PCN for 0.5, 1, 2, 4 and 8 h.

### Continuous confocal fluorescence microscope

The 1×10^5^ RAW 264.7 cells were seeded in a confocal dish and infected with MRSA at a ratio of 10 bacteria per cell. After 4 h of co-cultivation, 10 μg/mL PCN-224, GaPR-PCN and HA-GaPR-PCN were added to the sterile and MRSA-infected cells and incubated for 5-25 min (PCN-224 and GaPR-PCN) and 125-145 min (HA-GaPR-PCN), respectively. The nuclei of RAW 264.7 cells and MRSA were stained with 4,6-diamino-2-phenylindole (DAPI) for 15 min prior to observation. Intracellular dynamic transport of nanoparticles was observed using the CLSM (Nikon, N-STORM, Japan).

### Endocytic pathway analysis

The 2×10^5^ RAW 264.7 cells were seeded in 24-well plates and infected with MRSA at a ratio of 10 bacteria per cell. After infection, cells were treated for 4 h with chlorpromazine (3 μM), methyl-β-cytodextrin (4 mM), amiloride (25 μM), cytochalasin D (3 μM), anti-CD163 (50 μg/mL) and anti-LRP1 (50 μg/mL) for 0.5 h. After that, 10 μg/mL Lev-PCN-224, Lev-GaPR-PCN and HA-Lev-GaPR-PCN were added into the 24-well plates for co-incubation for 2 h, and then intracellular fluorescence was analyzed by flow cytometry.

### Effect of Hpx and Hpg on the cellular uptake of nanosystems

After successful infection of RAW264.7 cells with MRSA for 4 h, fresh medium or fresh medium containing 1 μg/mL Hpx and 4 μg/mL Hpg were used to replace the old medium. Subsequently, 10 μg/mL Lev-GaPR-PCN was added to RAW264.7 cells and incubated for 2 h. The amount of intracellular nanosystems was confirmed by flow cytometry via the determination of intracellular fluorescence to investigate the effect of Hpx and Hpg on the uptake of Lev-GaPR-PCN by RAW264.7 cells.

### Co-localization of nanosystems with intracellular MRSA

After successful infection of RAW 264.7 cells with MRSA for 4 h, 10 μg/mL PCN-224 and GaPR-PCN were added to the infected cells and co-incubated for 2 h. Cells were then rinsed with PBS, fixed with 4% paraformaldehyde for 15 min and treated with 5% fetal bovine serum for 1 h at 25 °C. The cells were then treated with an anti-*S. aureus* antibody (5 μg/mL) for 16 h at 4 °C. Fluorescently labeled secondary antibodies were then added for 2 h at 37 °C. Co-localization of intracellular nanosystems and MRSA was observed using the CLSM (Nikon, N-STORM, Japan).

### Co-localization of nanosystems with lysosomes and Golgi apparatus

RAW 264.7 cells were first treated with 10 μg/mL GaPR-PCN for 3 min, 15 min, 30 min, 1 h, 2 h and 4 h, respectively. Subsequently, the lysosomes and Golgi apparatus were stained with Lyso-Tracker Green and Golgi-Tracker Green (Beyotime, Shanghai, China) for 20 min. Co-localization of intracellular GaPR-PCN and subcellular organelles was observed using CLSM (Nikon, N-STORM, Japan).

### CD163 and LRP1 expression level detection on the membranes of sterile and infected cells

The immunofluorescence method was used to detect the expressed discrepancy of CD163 and LRP1 on the sterile and MRSA infected RAW264.7 cells for 24 h. The sterile and infected cells were fixed in 4% polyformaldehyde for 15 min and treated with 5% fetal bovine serum for 1 h at 25°C, followed by staining with anti-CD163 or anti-LRP1 antibody (5 μg/mL) for 16 h at 4°C. Subsequently, the CY3 labeled secondary antibodies (10 μg/mL) were added for 2 h at 37°C. The nuclei of sterile and infected cells were stained with DAPI. The cell surface fluorescence was measured by flow cytometry. The CD163 and LRP1 express level change with time were detected as above at the different time points after MRSA infections. All the experiment was independently done for 3 times.

### *In vivo* target performance, therapy effects and safety study of HA-Lev-GaPR-PCN

All animal experiments were conducted in strict accordance with the protocols approved by the Animal Care and Use Committee of Huazhong Agricultural University (Wuhan, China) under the approval number HZAUMO-2021-0186. The BALB/C mice were infected intramuscularly with 107 CFU MRSA on one hind leg overnight to establish the infection models. The infected mice (n=3) were injected with Lev-PCN-224, Lev-GaPR-PCN or HA-Lev-GaPR-PCN via the tail vein. At fixed time points after infection of the different nanosystems via the tail vein, NIRF images were acquired using an IVIS Lumina imaging system (PerkinElmer, USA). At the end of the experiment, the infected tissue was harvested for TEM observation (JEOL JEM-2100F, Japan).

Thirty-six BALB/C mice with infected legs were randomly divided into 6 groups (HA-Lev-GaPR-PCN, Lev and saline with and without laser irradiation), 6 mice per group. The infected mice were injected intravenously with HA-Lev-GaPR-PCN, Lev and PBS once daily for 5 consecutive days. The dose calculated as Lev was 36 mg/kg, except in the PBS group. After 4 h of intravenous administration, the infected area of the mice was irradiated twice daily for 10 min with a 660 nm laser at a power of 100 mW/cm2. The interval between the two irradiations was 30 min on one day. The number of MRSA and the pro- and anti-inflammatory factors in the infected legs were determined after the collection of tissue homogenates at the end of the experiment. The safety study was described in detail in the SI Appendix.

## Results and Discussion

### Design and screening of nutritional trojan horses

**Figure 2:**
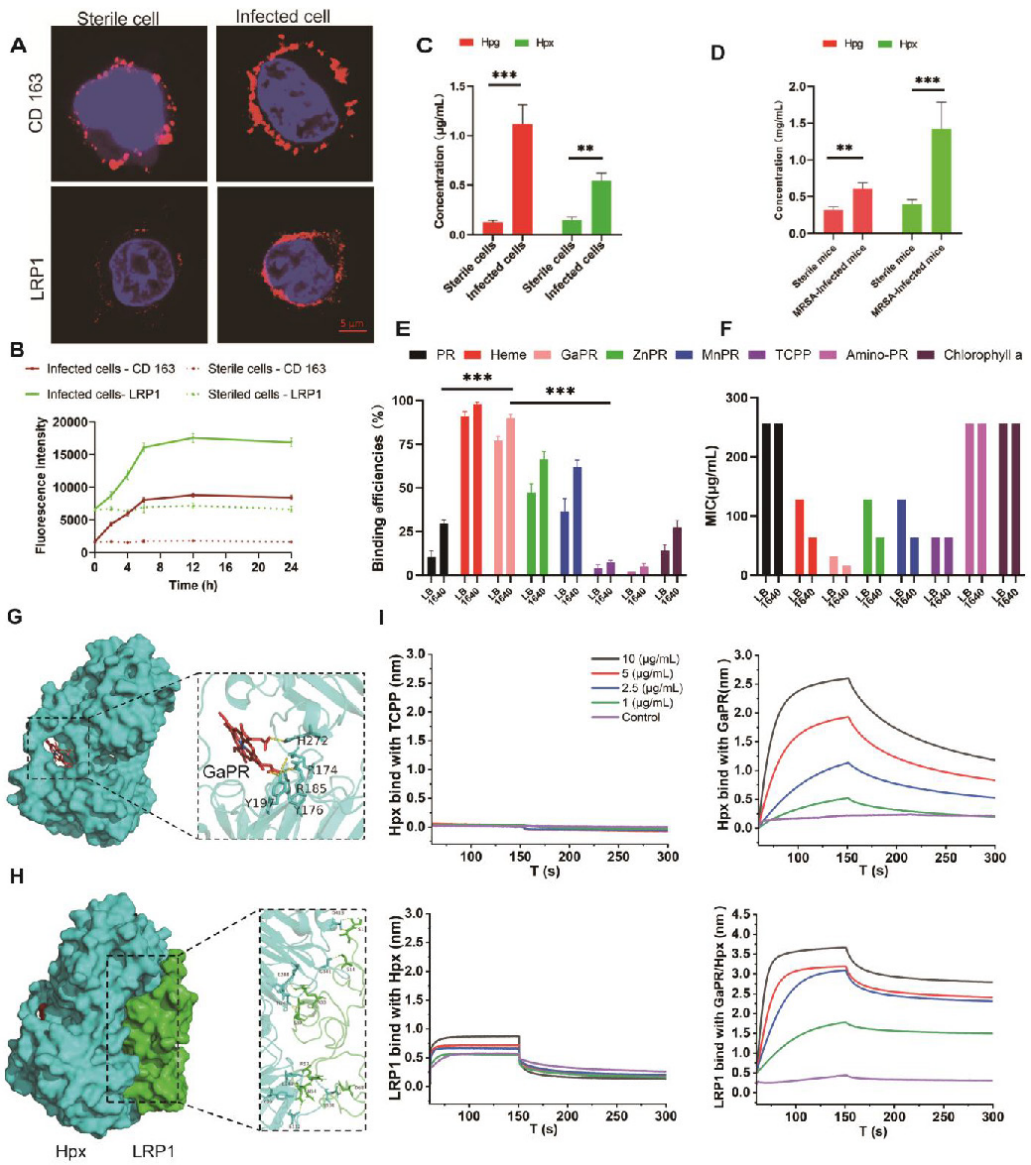
** Design and screening of targeting nutritional competitive antibiotics (trojan horse). (A-B)** Distribution of CD163 and LRP1 on the surface of sterile and MRSA-infected RAW 264.7 cells examined by CLSM **(A)** and flow cytometry **(B)**. **C)** Hpg and Hpx levels in sterile and MRSA-infected cell culture medium (n=3). **(D)** Hpg and Hpx levels in the plasma of healthy mice and MRSA-infected mice (n=3). **(E)** Binding efficiency of various porphyrin molecules with MRSA (n=3/group). **(F)** MIC of various porphyrin molecules against MRSA. **(G)** Three-dimensional ligand-protein interaction mode for the binding site of Hpx with the GaPR. **(H)** Three-dimensional ligand-protein interaction mode for the binding site of Hpx with LRP1. **(I)** Binding efficiency of TCPP and GaPR with Hpx and LRP1 determined by BLI.*p < 0.05; **p < 0.01 and ***p < 0.001.

The overexpression of the CD163 and LRP1 receptors in MRSA-infected RAW 264.7 macrophages was first confirmed. Confocal laser scanning microscopy (CLSM) examination revealed significantly greater red fluorescence intensities for CD163 and LRP1 in infected cells than in uninfected controls **(Figure 2A)**. Flow cytometry assays were performed to further quantify this upregulation, and the results revealed a time-dependent increase in receptor expression that correlated with the duration of infection **(Figure 2B)**. Moreover, higher levels of Hpg and Hpx proteins were secreted in the supernatants of MRSA-infected RAW 264.7 cells and in the plasma of infected mice, which were significantly greater than those in sterile cultures and healthy controls **(Figures 2C and 2D)**.

Using protoporphyrin IX dimethyl ester as a scaffold, a series of heme-mimetic porphyrins were synthesized by chelating gallium, manganese, or zinc ions **(Figure S1A)**. The results of flow cytometry assays revealed that GaPR was the most efficient MRSA binder, with >95% of the MRSA cells adsorbing GaPR or heme **(Figure 2E)**. Antibacterial activity testing showed the potent anti-MRSA activity of GaPR, with an MIC of 32 μg/mL in standard medium and an MIC of 16 μg/mL in iron-deficient culture medium **(Figure 2F)**. The increased efficacy in iron deficiency agrees with the "Trojan horse" mechanism of GaPR: the physicochemical mimicry of iron by gallium allows competitive uptake by bacterial iron-sensing systems and interferes with iron-dependent metabolism. The results of flow cytometry assays confirmed increased binding of GaPR-MRSA under iron-limited conditions **(Figure 2E)**, highlighting the utility of GaPR in iron-limited microenvironments by the host. Finally, the high binding efficiency of GaPR to the proteins secreted by Hpg and Hpx as well as to the receptor proteins CD163 and LRP1 was confirmed via interference at the biological level. GaPR bound specifically to Hpx with a KD of 0.799 μM, while TCPP did not bind. Premixing GaPR with Hpx increased the binding affinity (KD, 44.7 μM) for LRP1-overexpressing infected cells. Similar binding of GaPR with Hpg and increased interactions between CD163 and Hpg were observed **(Figure S1B).** These results suggested that GaPR is an efficient dual-purpose agent: a "Trojan horse" that interferes with bacterial iron uptake and a targeting ligand for infection-specific receptors. Owing to its selectivity, bactericidal efficacy, and synergy with host nutritional immunity, GaPR is a promising candidate against intracellular MRSA.

### Design, characterization, and programmed release of triple-targeting nanosystems

**Figure 3:**
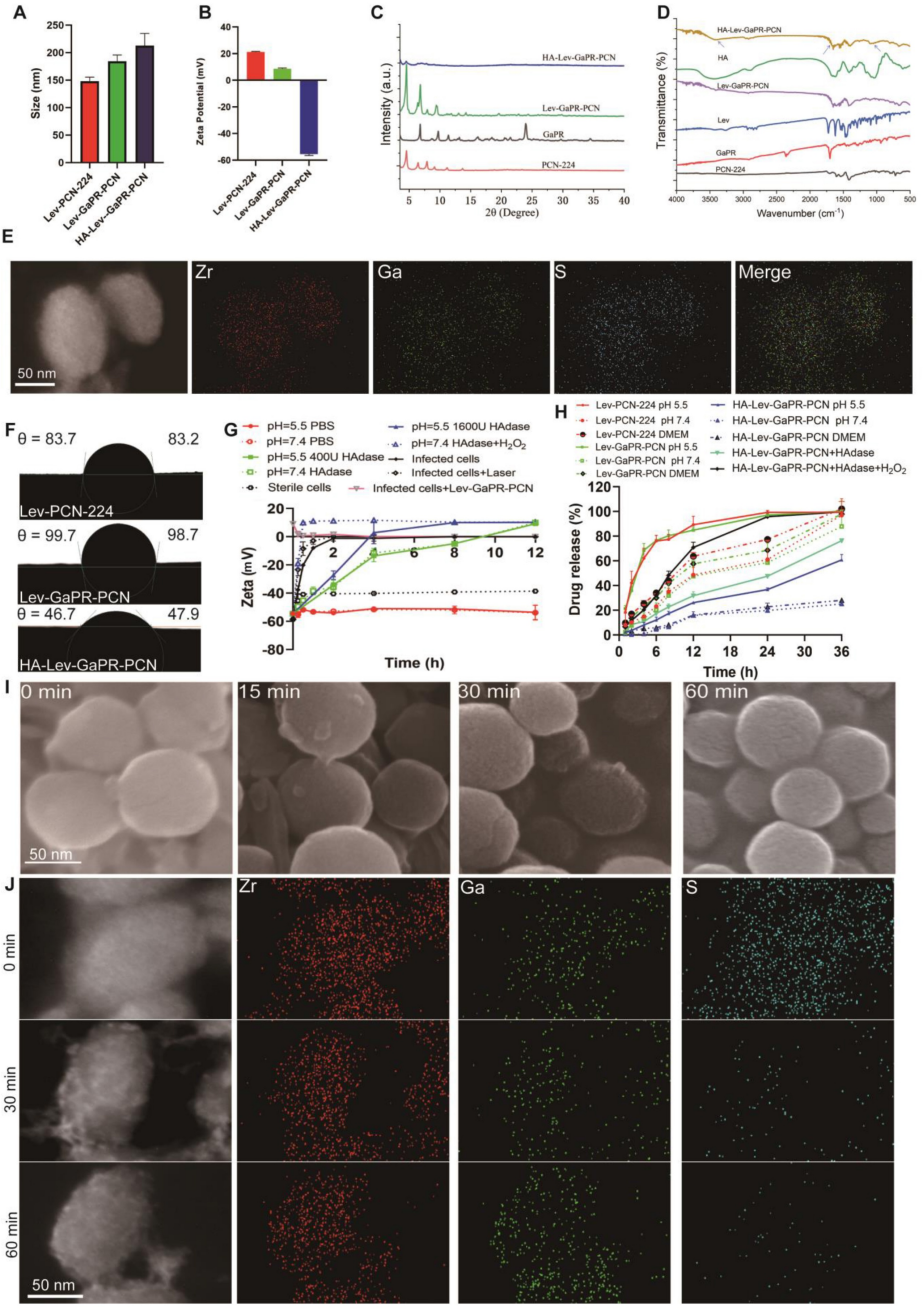
** Characterization and programmed release of different nanosystems. (A)** Dynamic light scattering (DLS) size of Lev-PCN-224, Lev-GaPR-PCN, and HA-Lev-GaPR-PCN. **(B)** Zeta potential of Lev-PCN-224, Lev-GaPR-PCN, and HA-Lev-GaPR-PCN. **(C)** PXRD pattern of Lev-PCN-224, GaPR, Lev-GaPR-PCN, and HA-Lev-GaPR-PCN (graph changed to arbitrary unit). **(D)** Fourier transform infrared spectrum (FT-IR) of PCN-224, GaPR-PCN, HA, and HA-Lev-GaPR-PCN. **(E)** TEM elemental mappings of HA-Lev-GaPR-PCN. **(F)** Water contact angle images of Lev-PCN-224, Lev-GaPR-PCN, and HA-Lev-GaPR-PCN. **(G)** Surface charge of HA-Lev-GaPR-PCN placed in PBS (pH=5.5/7.4), HAdase solution (400U), and H_2_O_2_ (50mM) solution, as well as incubating with sterile cells and MRSA-infected cells. **(H)** Lev release profiles from Lev-PCN-224, Lev-GaPR-PCN, and HA-Lev-GaPR-PCN in different pH PBS with and without HAdase and H_2_O_2_. **(I)** SEM images of HA-Lev-GaPR-PCN incubating with MRSA infected cells at different time points. **(J)** TEM elemental mappings of HA-Lev-GaPR-PCN treated with MRSA infected cells for different time points.

The triple-targeted HA-Lev-GaPR-PCN nanosystem, which consists of a Lev-loaded zirconium-based porphyrinic MOF coated with HA via TK linkers, was synthesized by adopting a multistep process **(Figure 1)**. Based on established protocols for multicomponent MOF synthesis 35, GaPR-PCN was prepared by coordination between Zr(IV) clusters and carboxylate linkers of TCPP and GaPR. GaPR is a gallium-chelated heme mimetic compound that competes with TCPP to form Zr6 nodes with mixed ligands, resulting in a stable, spherical nanoscale MOF **(Figure 3A)**. To optimize the efficiency of cellular uptake and PDT 36, we adjusted the concentrations of organic ligands to control the particle size **(Figure S2A)** and achieved a diameter of 184.5 ±11.3 nm and a zeta potential of 9.3 ±0.2 mV for GaPR-PCN **(Figures 3A-B)**. In contrast, the control MOF PCN-224 (synthesized with TCPP only to demonstrate the function of GaPR) was smaller (148.2 ±7.3 nm) and had a greater surface charge (20.1 ±0.5 mV). X-ray diffraction (XRD) confirmed the coexistence of characteristic PCN-224 peaks (4.6°, 6.4°, 7.9°, 9.1°, 11.2°, and 13.7°) and GaPR-specific peaks (6.3° and 9.9°) in GaPR-PCN (Figure 3c). FT-IR results showed a redshift at 1650 cm^-1,^ indicating carboxylate-Zr coordination with GaPR **(Figure 3D)**. These results indicated that the synthesis of GaPR-PCN was successful. GaPR-PCN has binding sites for incorporating heterotypic linkers and provides ample space to incorporate Lev. Although the size and zeta potential of GaPR-PCN did not change considerably after incorporating Lev compared to those of GaPR-PCN, UV-Vis spectroscopy (300 nm peak) and quantitative analysis confirmed the encapsulation of Lev (11.7 ±0.4% loading) in the GaPR-PCN mesopores **(Figure S1C)**. Lev-GaPR-PCN was stable in buffer solution, and the adsorption of Hpg and Hpx proteins increased particle size in infected environments **(Figure S2D)**.

To prolong the cycle time and further improve targeted delivery to the sites of MRSA infection, hydrophilic HA was linked to Lev-GaPR-PCN via a thioketal bond. The carboxyl groups on the free ligands in Lev-GaPR-PCN frameworks reacted with the amino groups of HA-TK-NH_2_ molecules. 1-Ethyl-3-(3-dimethylaminopropyl)carbodiimide/N-hydroxy succinimide (EDC/NHS) chemistry was used to activate carboxyl groups (-COOH) present on the edges of Lev-GaPR-PCN 37-38. The HA coating was visible on the surface of the nanoparticles via TEM examination **(Figure S2I)**. The altered size and zeta potential also indicated that the coating was successful. The reduced intensity of the characteristic peak of HA-Lev-GaPR-PCN **(Figure 3C)** indicated that the crystallinity decreased. The successful linkage of HA was also evidenced by the fact that the FT-IR absorption peaks at 1000-1100 cm^-1^, 1700 cm^-1^, and 3200-3600 cm^-1^ were from R-S-R' of the thioketal, the amide bond of HA, and the -OH of HA, respectively **(Figure 3D)**. The water contact angle data revealed that the hydrophilicity of HA-GaPR-PCN was significantly greater than that of GaPR-PCN **(Figure 3F)**. Maximising HA modification increases the cycle time and target efficiency of the nanosystem. As the content of HA-TK-NH2 increases, the size of HA-Lev-GaPR-PCN gradually increases. 40 mg HA-TK-NH2 modified HA-Lev-GaPR-PCN can hardly be taken up by normal cells within 2 h **(Figures S2B and C)**. The singlet oxygen inside and outside the cell generated by Lev-GaPR-PCN was attenuated by the HA shell under laser irradiation **(Figures S2G and H)**. TEM elemental mapping of HA-GaPR-PCN revealed a uniform distribution of Zr, S (TK-NH_2_), and Ga (GaPR) **(Figure 3E)**, which provided further evidence of effective GaPR deposition and HA coating. The modification of HA increases the stability of HA-Lev-GaPR-PCN **(Figures S2E and F)**.

The HA coating by TK bond should also serve as a barrier to prevent leakage of Lev during transport and improve targeting by reducing endocytosis by normal host cells. In MRSA-infected micro-environments (elevated HAdase and ROS levels 39-42), HA-Lev-GaPR-PCN underwent programmed activation to release the intact Lev-GaPR-PCN core and selectively target the secreted proteins of infected cells via surface GaPR at infection sites to more effectively discriminate infected cells. The degradation of the HA envelope was monitored by changing the surface charge after incubation with HAdase, hydrogen peroxide, and MRSA-infected cells **(Figure 3G)**. The surface potential shifted from -18.2 mV (HA-coated) to +9.1 mV (Lev-GaPR-PCN core) within 0.25 h under HAdase/H_2_O_2_ combination treatment **(Figure 3G)**. TEM/SEM examination revealed detachment of the HA shell and restructuring of the porous surface after incubation with HAdase-infected or MRSA-infected cells **(Figure S2I and Figure 3I)**. Laser irradiation (660 nm) accelerated the detachment of HA by increasing ROS formation **(Figures S2G-H)**. When MRSA-infected cells were exposed, the surface charge progressively decreased as the incubation time increased, and the intensity of S rapidly disappeared **(Figure 3J).** These results suggested that HA-Lev-GaPR-PCN can be programmed to release intact Lev-GaPR-PCN when it reaches infected sites.

*In vitro* release studies revealed that HA-Lev-GaPR-PCN released 16.8-27.3% Lev within 12 h in PBS solutions at different pH, whereas Lev-GaPR-PCN released a greater percentage of Lev (49.7-70.1%) within the same time frame. These results highlighted the high stability of HA-Lev-GaPR-PCN under physiological conditions, which effectively prevents the premature release of Lev during transport. At pH 5.5, in the presence of HAdase (400 U/mL) and H_2_O_2_ (50 mM), the release of Lev from HA-Lev-GaPR-PCN increased significantly to 78.4% within 12 h, whereas it was only 27.3% in PBS at pH 7.4, showing dual-responsive release kinetics **(Figure 3H).**

### Targeting performance and mechanism of nanosystems to MRSA

**Figure 4:**
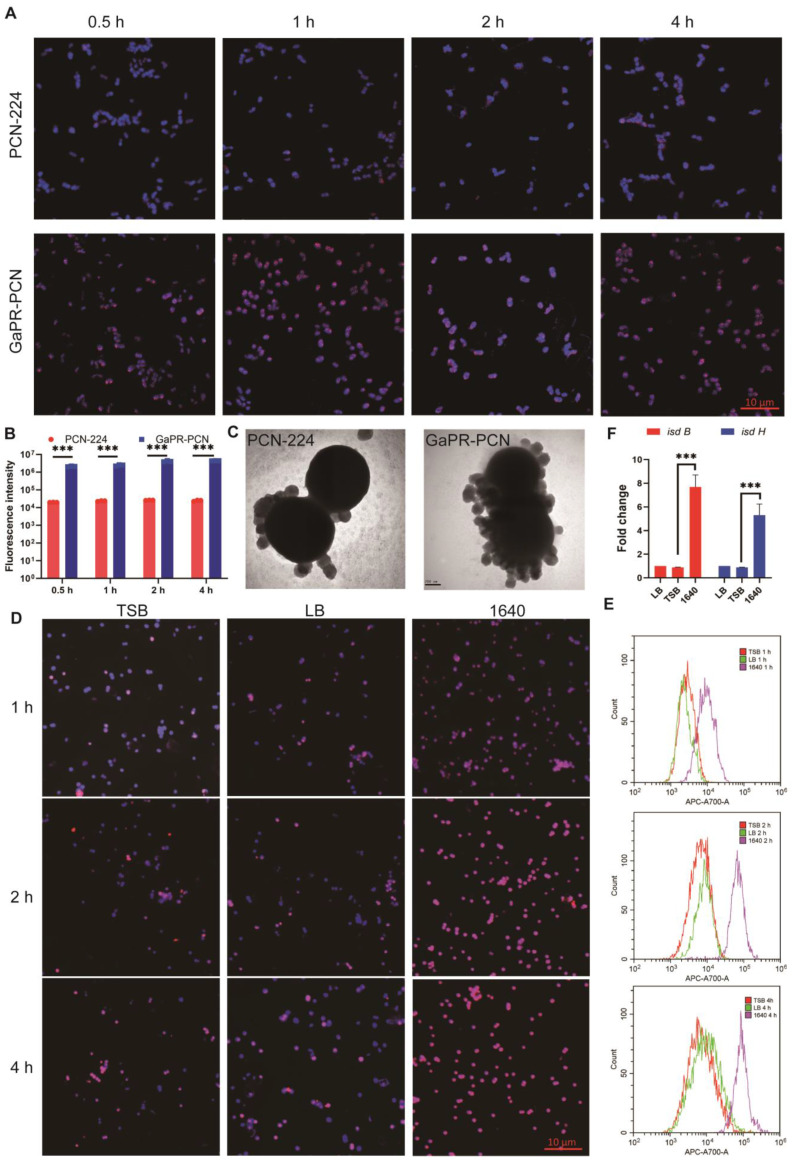
** Targeting performance and mechanism of HA-GaPR-PCN to MRSA. (A)** Fluorescence microscope images of PCN-224 and GaPR-PCN combined with MRSA. **(B)** Fluorescence counts of MRSA after co-incubating with PCN-224 and GaPR-PCN for 0.5-4 h. **(C)** TEM images of PCN-224 and GaPR-PCN combined with MRSA for 2h.** (D)** Fluorescence microscope images of PCN-224 and GaPR-PCN combined with MRSA in TSB, LB, and 1640 media for 1-4 h. **(E)** Flow cytometry images of APC-A700 fluorescence counts of MRSA after co-incubating with PCN-224 and GaPR-PCN in TSB, LB, and 1640 media for 1-4 h.** (F)** RT-qPCR quantification of isdb and isdh expression (n=6/group) levels.

The ability of GaPR-PCN to combat MRSA was first investigated via fluorescence microscopy. After coincubation for 1 h, strong colocalization was observed between the blue fluorescent MRSA cores (DAPI staining) and the red fluorescent GaPR-PCN cores **(Figures 4A and B)**, indicating efficient binding of the bacteria. The interactions between the nanosystem and MRSA were subsequently quantified via flow cytometry assays. After 15 and 30 min, 50.32-90.54% of the MRSA strains were fluorescence-positive for GaPR-PCN, whereas 18.14-42.65% were fluorescence-positive for PCN-224 (control MOF without GaPR) **(Figure 4B; Figure S2J)**. MRSA treated with GaPR-PCN presented a 2.11-3.32-fold greater signal than bacteria treated with PCN-224, highlighting the role of GaPR in enhancing adhesion (Figure 4b). TEM images confirmed the above results and revealed dense GaPR-PCN aggregates on MRSA surfaces compared to sparse PCN-224 adsorption **(Figure 4C)**. To determine whether GaPR-PCN binding is mediated by the heme acquisition Isd system of MRSA, we compared the binding efficiency under iron-rich (TSB medium) and iron-poor (RPMI-1640 medium) conditions 43. The binding kinetics of GaPR-PCN with MRSA in low-iron media (1640) were about three times greater than those in iron-rich media (TSB) after 1 h **(Figures 4D-E)**. The qRT-PCR assays revealed a 6.2-9.0-fold greater expression of isdB and isdH, genes encoding heme-binding proteins of the Isd system, in MRSA cultured in 1640 than in those cultured in TSB **(Figure 4F)**. The results showed that GaPR-PCN acts selectively on MRSA by targeting the Isd system, with enhanced binding efficacy under iron-limiting conditions that mimic host nutritional immunity. The IsdB/IsdH proteins probably serve as primary receptors for GaPR-mediated adhesion.

### Targeting performance and mechanism of nanosystems for infected cells and intracellular MRSA

**Figure 5:**
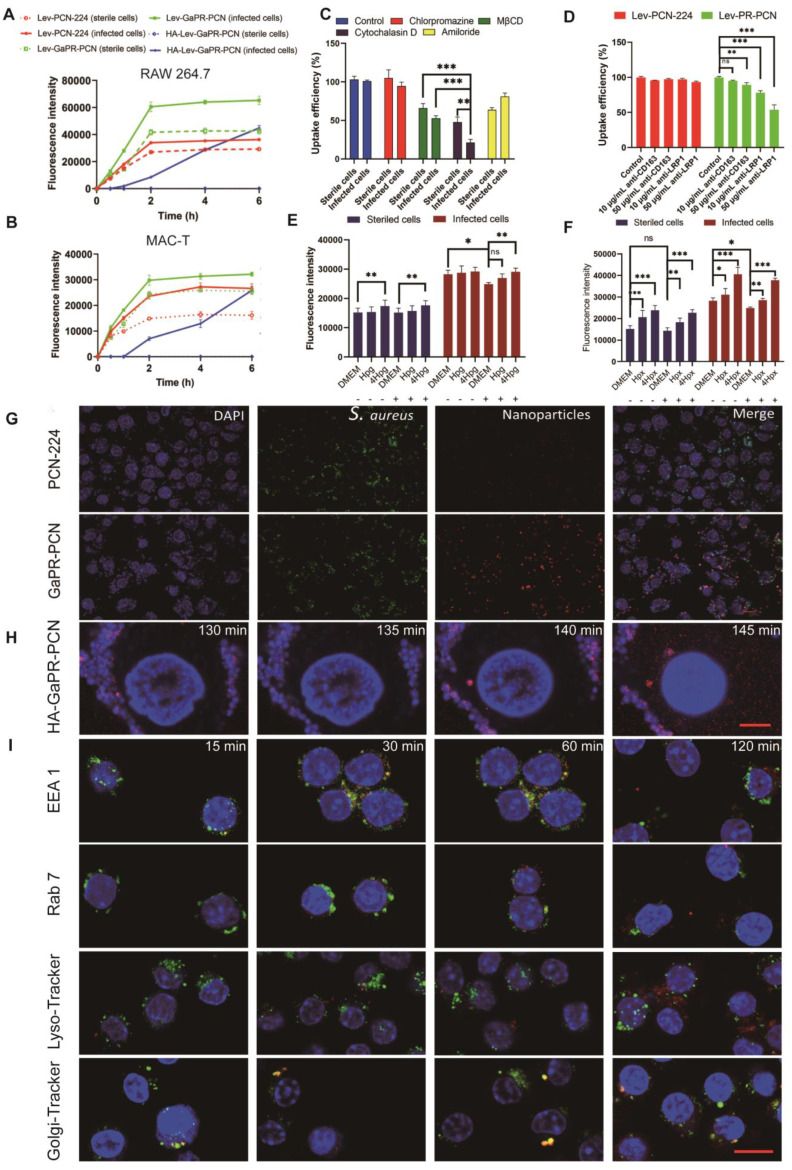
** Targeting performance and mechanism of nanosystems to infected cells and intracellular MRSA. (A-B)** Cellular uptake of PCN-224, GaPR-PCN, and HA-GaPR-PCN by sterile and MRSA-infected RAW264.7 **(A)** and MAC-T cells **(B)** (n=3). **(C, D)** Cellular uptake inhibition of Lev-GaPR-PCN by RAW 264.7 after 2 h of co-incubation of CPZ (chlorpromazine), CYT (Cytochalasin), AMI (amilori) and anti-CD163 and anti-LRP1. **E-F)** Cellular uptake of Lev-GaPR-PCN by sterile and MRSA-infected RAW 264.7 cells when removing Hpx **(E)** and Hpg **(F)** by the fresh culture medium and spiked Hpg and Hpx into old culture medium. Replaced culture medium (-), without replacing the medium (+). **(G)** Intracellular co-localization of PCN-224 and GaPR-PCN with MRSA determined by CLSM. Scale bars, 10 μm. **(H)** Continuous confocal fluorescence microscope images of HA-GaPR-PCN transport into MRSA infected RAW 264.7 cells during 125-145 min. Scale bars, 5 μm. **(I)** Intracellular co-localization of GaPR-PCN with early endosomes, late endosome, Golgi apparatus, and lysosome. Scale bars, 10 μm.

Fluorescence microscopy revealed different uptake patterns of the nanosystems between sterile and MRSA-infected cells. GaPR-PCN and the control PCN-224 had significantly greater fluorescence intensities than their sterile counterparts in infected RAW 264.7 macrophages and nonphagocytic bovine epithelial cells (MAC-T) after the same incubation time (Figures 5a and b). GaPR-PCN showed 1.8-fold to 2.5-fold greater intracellular fluorescence than PCN-224, which can be attributed to the targeting functionality of GaPR. HA-Lev-GaPR-PCN, with its hydrophilic HA coating (surface charge: -60 mV; contact angle: 46.7°), showed minimal internalization by sterile cells (< 5% fluorescence intensity at 6 h) but progressive accumulation in infected cells (> 60% after 1 h) **(Figure 5A)**. This differentiation occurred due to the degradation of HA by MRSA-secreted HAdase and ROS at infection sites, which exposed the hydrophobic and positively charged GaPR-PCN core (+9.3 mV) for selective receptor-mediated uptake. This phenomenon contributes to their long persistence in the body, as they evade phagocytosis by normal host cells and selectively attach to infected cells. CLSM also confirmed that a substantial number of GaPR-PCNs (better than PCN-224) were internalized by MRSA-infected RAW 264.7 cells **(Figure S3A)**. In contrast, only a tiny fraction was present in sterile cells** (Figure S3B)**.

To elucidate the expected targeting mechanism for infected cells, the endocytic pathways were first investigated. Pretreatment with MβCD (lipid raft inhibitor) and cytochalasin D (actin polymerization blocker) decreased the uptake of Lev-GaPR-PCN by 34.0% and 47.2% (sterile/infected cells) and 52.2% and 78.7%, respectively **(Figure 5C).** These results indicated that the nanosystems are internalized by lipid raft-mediated and receptor-mediated endocytosis. Lipid raft-mediated endocytosis can bypass lysosomes, which is consistent with the subcellular distribution of Lev-GaPR-PCN and may facilitate its escape from lysosomal degradation 44. We performed further studies to determine whether the enhanced receptor-mediated endocytosis of Lev-GaPR-PCN occurred via the overexpressed heme acquisition systems in infected RAW 264.7 cells, as hypothesized. Depletion of Hpx/Hpg in the medium of infected cells decreased the uptake of Lev-GaPR-PCN by 12.0%, whereas uptake by sterile RAW 264.7 cells remained unchanged. Uptake of Lev-GaPR-PCN by healthy and infected RAW 264.7 cells was restored and increased in a dose-dependent manner **(Figures 5E and F)**. The cellular uptake of Lev-GaPR-PCN increased by 1.3-1.6-fold in healthy cells and by 1.2-1.4-fold in infected cells when the concentration of restored Hpx increased from 1 to 4 μg/mL **(Figure 5E)**. The uptake of Lev-GaPR-PCN by healthy RAW 264.7 cells increased by 14.3% after 4 μg/mL Hpg **(Figure 5F)** was added. Competitive inhibition with heme (an Hpg/Hpx antagonist) confirmed that Hpg and Hpx are crucial mediators of targeting **(Figures S3G-H)**. To further characterize the receptor-specific contributions, the cellular uptake of nanosystems by MRSA-infected RAW 264.7 cells was investigated after antagonizing the CD163 and LRP1 receptors with their respective antibodies. Treatment with 10 μg/mL and 50 μg/mL LRP1 antibodies reduced the cellular uptake of Lev-GaPR-PCN by 21.9% and 46.2%, respectively **(Figure 5D)**. A 50 μg/mL dose of CD163 antibody reduced the intracellular Lev-GaPR-PCN level by 10.7% **(Figure 5D)**. These results showed that both the LRP1 and CD163 receptors contribute to the internalization of nanosystems, with LRP1 playing a dominant role in infected macrophages. Additionally, BLI was used to confirm the binding and dissociation of GaPR-PCN with the heme acquisition systems Hpx/LRP1 and Hpg/CD163, respectively. Premixing GaPR-PCN with Hpx increased the binding affinity to LRP1 (KD, 48.0 μM vs. 0.863 μM for GaPR-PCN alone; **Figure S3C**). A similar increase in CD163 was found when GaPR-PCN was premixed with Hpg, although the binding was weaker **(Figure S3D)**. These results confirmed that Hpx and Hpg act as molecular bridges and facilitate the recognition of overexpressed LRP1 and CD163 in infected cells by GaPR-PCN.

To assess the intracellular trafficking and MRSA-targeting performance of GaPR-PCN, GaPR-PCN was used to exclude the effect of Lev on the activity and abundance of MRSA. GaPR-PCN showed 3.42-fold greater colocalization with intracellular MRSA (Pearson correlation coefficient (r) = 0.41) than PCN-224 (r = 0.12) **(Figure 5G)**. Real-time confocal imaging revealed dynamic targeting: GaPR-PCN associated with MRSA within 5 min and colocalized almost completely after 25 min **(Figure S3E)**. The colocalization of HA-Lev-GaPR-PCN was delayed by HA shielding and showed analogous behavior after the degradation of HA. Similar observations were made in MAC-T cells **(Figure 5H)**. To visualize intracellular transport, anti-EEA1, anti-Rab 7, anti-Rab 6, LysoTracker Green, and Gly-Tracker Green were used to examine colocalization with lysosomes and the Golgi apparatus (Figure 5d). After 15 min of incubation, a substantial amount of Lev-GaPR-PCN was colocalized with early endosomes, whereas only minimal colocalization was observed in late endosomes. Within 4 h, Lev-GaPR-PCN showed minimal colocalization with lysosomes (r < 0.15) **(Figure 5I)**. However, a significant portion of Lev-GaPR-PCN was colocalized with Rab6 and the Golgi apparatus after 15 min of coincubation, suggesting that Lev-GaPR-PCN is transported via the trans-Golgi pathway **(Figure S3F)**. This nonlysosomal pathway bypasses lysosomal degradation and preserves the integrity of the nanosystem, allowing sustained antibacterial activity in the cytoplasm where MRSA resides 45.

### Triple-target performance and safety of nanosystems *in vivo*

**Figure 6:**
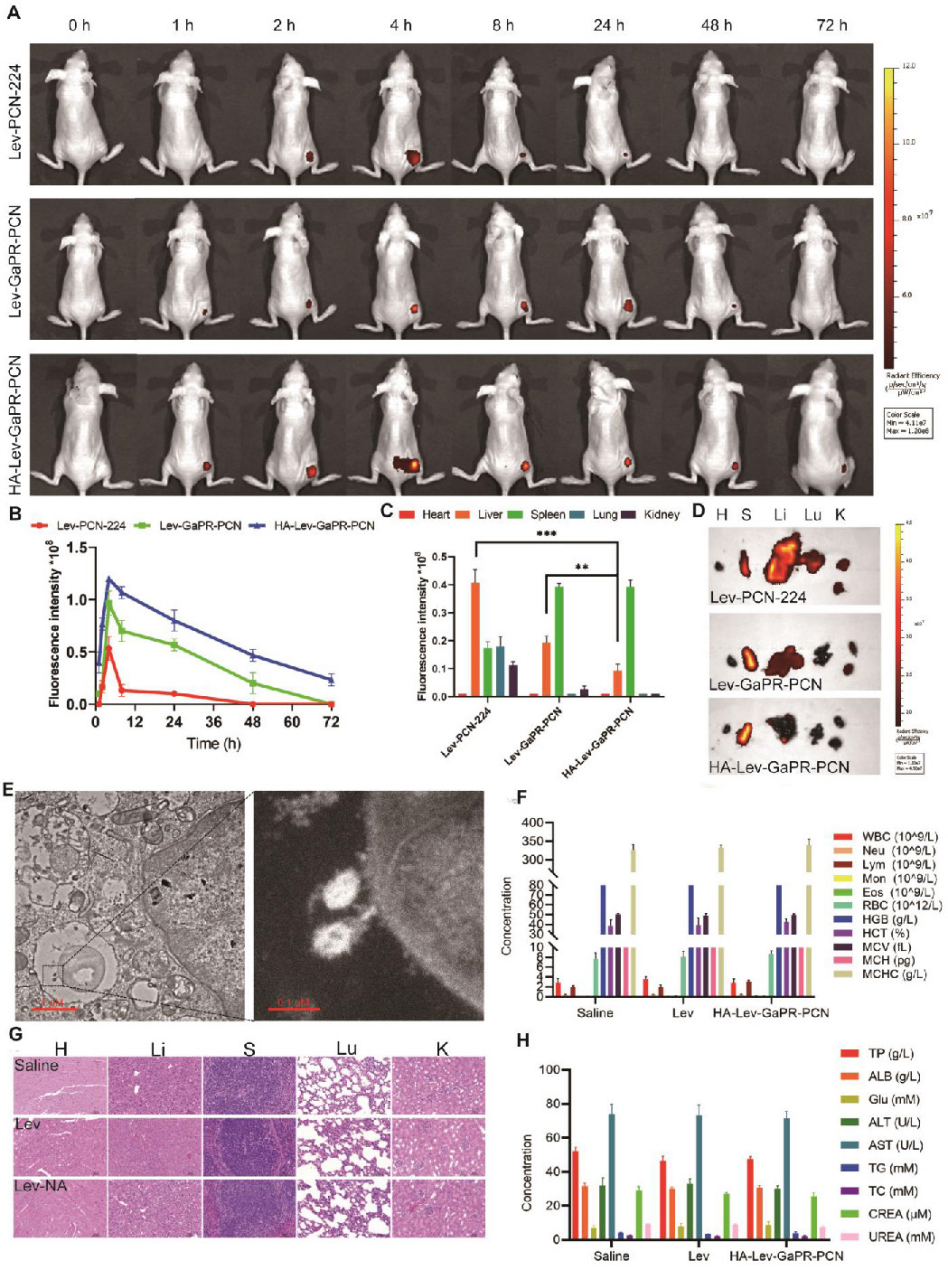
** Multiple target performances and safety of nanosystems *in vivo*. (A)** IVIS images of MRSA infected subcutaneous abscess mice after tail vein injection of Lev-PCN-224, Lev-GaPR-PCN, and HA-Lev-GaPR-PCN over 72 h (n = 3). **(B)** Quantitative analysis of fluorescence intensity in the legs of mice from each group (n = 3). **(C)** Fluorescence intensities of the major organs at 72 h (n = 3). **p < 0.01, ***p < 0.001. **(D)** Fluorescence imaging in the major organs after intravenous injection of Lev-PCN-224, Lev-GaPR-PCN, and HA-Lev-GaPR-PCN at 72 h (n = 3), respectively. Heart (H), liver (Li), spleen (S), lungs (Lu), kidneys (K). **(E)** TEM images of co-localization of HA-Lev-GaPR-PCN with intracellular MRSA. Scale bars, 1 μm. **(F)** Hematological parameters of WBC, Neu, Lym, Mon, Eos, RBC, HGB, HCT, MCV, MCH, and MCHC in three different treatment groups (n=5). **(G)** H&E staining of major organs sections of mice after saline, Lev, and HA-Lev-GaPR-PCN (Lev-NA) treatment for 14 d. Heart (H), liver (Li), spleen (S), lungs (Lu), kidneys (K). **(H)** Biochemical parameters (TP, ALB, Glu, ALT, AST, TG, TC, CREA, and UREA) in three different treatment groups (n=5).

To evaluate the triple-targeting ability of the nanosystems, Lev-PCN-224, Lev-GaPR-PCN, and HA-Lev-GaPR-PCN were administered intravenously to mice with subcutaneous MRSA abscesses. Lev-GaPR-PCN and HA-Lev-GaPR-PCN localized to infection sites within 2 h of injection and peaked after 4 h **(Figure 6A)**. Compared to Lev-GaPR-PCN (48 h) and Lev-PCN-224 (24 h), HA-Lev-GaPR-PCN presented a longer residence time (72 h) **(Figure 6A)**. HA-Lev-GaPR-PCN showed a 1.5-2.5-fold greater fluorescence intensity at the infection sites than Lev-GaPR-PCN from 8 to 72 h **(Figure 6B)**. The enhanced targeting of Lev-GaPR-PCN may be due to its effective binding to the highly secreted Hpx and Hpg proteins at infected sites. The hydrophilicity of HA and dual release (HAdase/ROS) prolonged circulation and enhanced infection-specific accumulation.

Moreover, Lev-PCN-224 and Lev-GaPR-PCN were mainly distributed in the liver, whereas HA-Lev-GaPR-PCN was mainly distributed in the spleen **(Figures 6C and D)**. This distribution pattern occurred probably due to the hydrophilic HA envelope, which reduces nonspecific phagocytosis in the liver. TEM examination of infected muscle tissue confirmed the colocalization of the nanosystem with intracellular MRSA **(Figure 6E)**, indicating that the nanosystem was intracellularly targeted. To evaluate the safety of HA-Lev-GaPR-PCN with laser irradiation, a study was conducted in healthy mice that were continuously administered HA-Lev-GaPR-PCN for 14 days. Daily administration of HA-Lev-GaPR-PCN to healthy mice caused no mortality, no abnormalities in hematologic/blood biochemical parameters **(Figures 6F and H)**, and no inflammatory lesions (histopathology; **Figure 6G**). These results showed that HA-Lev-GaPR-PCN does not cause subacute toxicity and is extremely safe due to its high targeting accuracy. The targeting accuracy and safety support the clinical application of this approach against persistent MRSA infections.

### Synergistic therapeutic effects of HA-Lev-GaPR-PCN nanosystems

**Figure 7:**
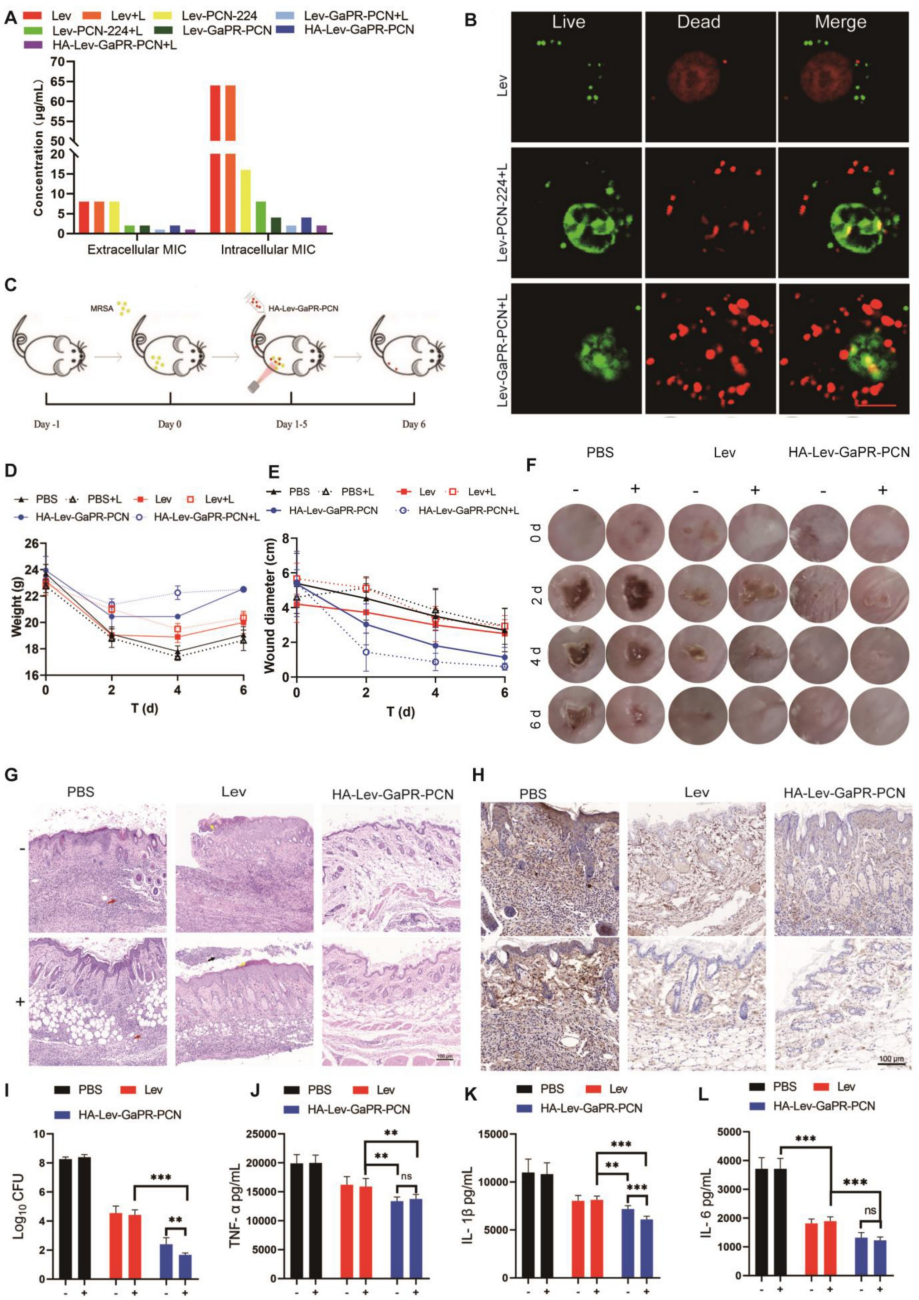
** Synergistic therapy effects of HA-Lev-GaPR-PCN nanosystems. (A)** Extracellular and intracellular MIC of three nanosystems and free Lev against MRSA with and without laser irradiation. **(B)** Confocal images of intracellular live and dead MRSA treated with 8 μg/mL Lev, Lev-PCN-224 and Lev-GaPR-PCN, respectively. **(C)** Treatment schematic of Lev and HA-Lev-GaPR-PCN for mice subcutaneous abscess. **(D)** Body weight of mice during experiment period (n=6). E) Diameter of mice abscesses during experiment period (n=6). **(F)** Local images of abscess site in mice during experiment period (n=6). **(G)** H&E staining of infected skin tissues at the end of experiment (n=6). **(H)** Fluorescence microscope images of TNF-α in mice after different treatments for 6 d. **(I)** Bacteria CFU in abscess homogenates in different treated groups at end of the experiment. **(J-L)** TNF-α, IL-1β, and IL-6 levels in abscess homogenates at the end of experiment. +, Laser irradiation; -, No laser irradiation. *p < 0.05; **p < 0.01 and ***p < 0.001.

The results of cytotoxicity tests revealed that the safe PDT parameters were irradiation with a 660-nm laser for 10 min, followed by a 30-min break and a second round of irradiation for 10 min **(Figures S3I-K)**. Extracellular MIC assays revealed that PCN-224 loaded with Lev (without irradiation) had an MIC of 8 μg/mL, whereas Lev-GaPR-PCN decreased the MIC to 2 μg/mL, demonstrating the nutrient-blocking synergy of GaPR **(Figure 7A)**. Adding Lev to GaPR-PCN decreased the intracellular MIC from 64 μg/mL to 4 μg/mL (without irradiation). Under PDT, the extracellular and intracellular MICs further decreased to 1 μg/mL and 2 μg/mL (Lev equivalent), respectively **(Figure 7A)**. Live/dead staining confirmed these results, with nearly complete eradication of bacteria observed in the Lev-GaPR-PCN + PDT group **(Figure 7B)**. This increase in efficacy occurred due to the targeted intracellular delivery of Lev and GaPR and the synergy between Lev (the inhibition of DNA gyrase), GaPR (the disruption of iron metabolism), and PDT (ROS-mediated damage).

The therapeutic efficacy was investigated in an acute subcutaneous MRSA abscess model in mice **(Figure 7C)**. Compared to the mice in the control group, those in the HA-Lev-GaPR-PCN + PDT group maintained a stable weight and performed better **(Figure 7D)**. On day 6 of treatment, the average diameter of the infection foci was 0.6, 1.1, 2.8, and 3.3 cm in the HA-Lev-GaPR-PCN group under laser irradiation, the HA-Lev-GaPR-PCN group without laser irradiation, the Lev group, and the PBS group, respectively **(Figures 7E and F)**. The bacterial load of the infected tissue showed that HA-Lev-GaPR-PCN + PDT decreased the number of MRSA colonies by 501-fold (2.7 log) compared to that of the Lev group, whereas the non-PDT group achieved a 63-fold (1.8 log) reduction **(Figure 7I)**. After the experiment ended, histopathological and cytokine analyses of the infected tissue were performed. HA-Lev-GaPR-PCN + PDT restored the near-normal skin architecture, whereas treatment with Lev led to neutrophil necrosis (**Figure 7G**, black arrow) and damage to the stratum corneum (yellow arrow). The PBS controls showed severe inflammatory infiltration (red arrow). The levels of proinflammatory cytokines (TNF-α, IL-6, and IL-1β) were significantly lower in the HA-Lev-GaPR-PCN + PDT group than in the PBS and Lev control groups **(Figures 7H and J-L)**. No subacute toxicity was found in healthy mice after 14 days of HA-Lev-GaPR-PCN administration with daily PDT. These results showed that HA-Lev-GaPR-PCN has excellent targeting ability and synergistic therapeutic efficacy against MRSA infections. By combining chemical agents (Lev), nutritional blockade (GaPR), and physical damage (PDT), HA-Lev-GaPR-PCN performs orthogonal antibacterial functions that suppress the development of resistance while avoiding the high cost of developing new drugs. This triple-modal strategy represents a sustainable, precise approach to combat intracellular multidrug-resistant infections.

## Conclusion

Most conventional antibiotics have low clinical therapeutic efficacy against intracellular MRSA due to their nonspecific biodistribution, inefficient cellular uptake, lysosomal degradation, and insufficient intracellular colocalization with bacteria. Additionally, conventional monotherapies (Lev, GaPR, or PDT) often easily lead to resistance. Therefore, we proposed an innovative triple-targeted and synergistic therapeutic strategy to combat intracellular MRSA infections using the common heme acquisition machinery of infected host cells and MRSA. By integrating GaPR, a multifunctional agent that serves as a synthetic recognition molecule and an "iron Trojan horse" into our nanosystem, we achieved triple targeting through three sequential steps: (1) Targeting the infection site: GaPR binds to host-derived heme-binding proteins of Hpg and Hpx enriched at infection sites. (2) Targeting infected cells: GaPR binds to Hpx and Hpg secreted at infection sites and improves the recognition of overexpressed LRP1 and CD163 receptors, respectively, on infected host cells. (3) Intracellular targeting of bacteria: Intracellular targeting directly interacts with the Isd of intracellular MRSA. After entering the cells, Lev-GaPR-PCN is transported via the trans-Golgi route, bypassing the lysosomes. This nonlysosomal route bypasses lysosomal degradation and preserves the integrity of the nanosystem, allowing efficient antibacterial activity in the cytoplasm where MRSA resides. To minimize off-target effects, the nanosystem is conjugated to HA via ROS-responsive TK binding, which ensures selective accumulation at infection sites while preventing recognition by uninfected cells. Moreover, GaPR has dual therapeutic functions. It is used as a "Trojan horse" to disrupt the iron metabolism of bacteria by mimicking heme and thus deprives pathogens of important iron resources. As photosensitizers, they generate bactericidal ROS when exposed to light. Thus, this nanosystem can exert synergistic antibacterial effects with encapsulated Lev (inhibition of DNA gyrase), GaPR (blockade of iron metabolism), and PDT (ROS-mediated damage) to eradicate MRSA through orthogonal mechanisms and effectively circumvent resistance. Integrating chemical, metabolic, and physical antibacterial methods not only eliminates the need to develop new antibiotics but also provides a blueprint for treating other resistant intracellular pathogens. This study represents a paradigm shift in antimicrobial therapy, offering a streamlined and clinically feasible solution to combat intracellular-resistant bacterial infections with high efficacy and specificity.

## Supplementary Material

Supplementary materials and methods, figures and table.
