# Supplementary material for: Triple-targeting nanosystems with synergistic effects on iron Trojan horse, fluoroquinolone antibiotics, and photodynamic therapy specifically kill intracellular MRSA
Source: Theranostics. 2025 Aug 16;15(17):9326–43. doi: 10.7150/thno.109374 (PMC12439474; doi:10.7150/thno.109374)
Supplement: Supplementary file 1 — Supplementary materials and methods, figures and table. [file thnov15p9326s1.pdf]

Triple-targeting nanosystems with synergistic effects on iron Trojan horse, fluoroquinolone antibiotics, and photodynamic therapy specifically kill intracellular MRSA

Kuiyu Meng[a,b], Liwen Yuan[c], Lulu Feng[a], Yaoyao Zhang[b], Hao Wu[b], Jie Zhang[b], Mubbashar Abbas[a], Wei Qu[c], Dongmei Chen[c], Shuyu Xie[a,b,c,d*]

[a]National Key Laboratory of Agricultural Microbiology, Huazhong Agricultural University, Wuhan, Hubei 430070, China

[b]Hubei hongshan Laboratory, Wuhan, Hubei 430070, China

[c]National Reference Laboratory of Veterinary Drug Residues (HZAU), Wuhan, Hubei 430070, China

[d]Key Laboratory of Prevention & Control for African Swine Fever and Other Major Pig Diseases, Ministry of Agriculture and Rural Affairs,Wuhan, Hubei 430070, China

*Corresponding author:

Professor Shuyu Xie, E-mail: Xieshuyu@mail.hzau.edu.cn, Tel: +86-27-87287323-8110, Fax: +86-27-87672232

Materials and Methods

Synthesis of GaPR

The protoporphyrin IX dimethyl ester (0.5 g) was dissolved in 30 mL of 2,6-lutidine and then added dropwise to 10 mL of 2,6-lutidine containing gallium chloride (4.93 g) under nitrogen addition. The mixture was reacted for 6 h at 150 ℃. After cooling, 500 mL of concentrated salt solution was added and the pH was adjusted to 4 with citric acid. The purple sediment was collected, washed with deionized water (3 x 100 mL) and identified by UV/vis.

Synthesis of HA-TK-NH_2

500 mg HA was dispersed in 500 mL pH 7 deionized water contained 310 mg 1-(3-Dimethylaminopropyl)-3-ethylcarbodiimide hydrochloride and 115 mg N-Hydroxysuccinimide. The mixture was stirred at 300 rpm for 4 h, and 194 mg HA-TK-NH_2 was added for continuous stirring for 24 h.The product was obtained by dialysis in deionized aqueous solution.

Size and zeta potential determination

The DLS and zeta potential were conducted by a Zetasizer Nano ZS ZEN3600 analyzer (Malvern Instrument, Britain). The zeta potential was obtained by measuring the electrophoretic movement of charged nanosystems under an applied electric field.

TEM observation

TEM images of different nanosystems were taken on a JEOL JEM-2100F (Japan) at an acceleration voltage of 200 keV by dropping the samples onto a

carbon-coated copper grid. The Image-Pro Plus image analysis software was used to measure the diameter of nanoparticles.

SEM observation

The nanosystems were adhered to the conductive adhesive and sprayed with gold with Quorum SC7620 for 45s. Then, the morphology was observed with TESCAN MIRA LMS (Czech) of scanning electron microscope.

Determination of $^1O_2$

10 μl of the 5 μM SOSG fluorescent probe methanol solution was added to 5 μl of Lev-PCN-224, Lev-GaPR-PCN and HA-Lev-GaPR-PCN suspension and then mixed uniformly with 85 μl of water. Under irradiation with a laser (660 nm) at different time points, the fluorescence intensity was measured using a multilabel microplate detection system (PerkinElmer Ltd., MA, USA) at an excitation/emission (Ex/Em) of 490/520 (SOSG). After successful infection of RAW264.7 cells with MRSA for 4 h, the cells were treated separately with 8 μg/mL of different nanosystems for 0.5-2 h. After 30 min of irradiation, the culture medium was washed off, SOSG was added and incubated at 37 °C for 30 min. The intracellular fluorescence was analyzed by flow cytometry (ex: 490 nm, em: 520 nm).

***In vitro* cytotoxicity**

Cells (5×103cells/well) were cultured in sterile 96-well plates for 24 h and treated with different concentrations of HA-Lev-GaPR-PCN (1.0, 2.0, 5.0, 10.0 and 20.0 μg/mL) for 4 h. They were then irradiated at 660 nm for 10, 10+10

(with a break of 30 min), 10+10+10 (with two breaks of 30 min), 20 and 30 min, respectively. The cells were then incubated for 24 hours after replacement with fresh culture medium before the cytotoxicity of the cells was checked using the MTT assay according to the manufacturer's protocol.

Bio-layer interferometry binding (BLI) kinetics assay

BLI was analyzed with the Fortebio red96e (PALL, USA) at an oscillation speed of 1000 rpm and a plate temperature of 30 °C. The biotinylation ligand protein was prepared according to the manufacturer's instructions using the kit (ARL0020S, Frdbio). Before testing the target analyte, the streptavidin light guide probe was placed in PBS for 10 min to load the biotinylation ligand (100 μg/mL) for 10 min. Then the probe was bound to different concentrations (10, 5, 2.5, 1, and 0 μg/mL) of analytes for 90 s to determine binding kinetics, followed by dissociation in PBS for 150 s.

Live/Dead cell staining assay

After successful infection of RAW264.7 cells with MRSA for 4 h, the cells were treated separately with 8 μg/mL Lev, Lev-PCN-224 and Lev-GaPR-PCN for 20 h. Then, the incubated cells were stained with a live/dead staining solution (Invitrogen, USA) (5 μM SYTO 9, 30 μM PI) for 20 min. Subsequently, the intracellular live/dead MRSA was observed using CLSM (Nikon, N-STORM, Japan).

qRT-PCR analysis

The MRSA at the concentration of 10^8 CFU was grown in 1640 and TSA medium under 220 rpm at 37 ° C for 4 h. The bacteria suspension was centrifuged at 12000 r/mim for 5 min, and then 100 μL of 2.5 μg/ml lysostaphin and 0.25 μg/ml lysozyme were added, respectively. The cell suspension was incubated at 37 ° C for 15 min and then extracted by RNeasy mini kit (Qiagen) according to the manufacturer's instructions. ImProm II cDNA Synthesis Kit (Promega) was used for RNA (50 ng/μL) generating cDNA. The referred primer sequences are shown in Table S1. The cDNA was analyzed with LightCycler 480 II real-time PCR instrument and SYBR reagent (Bio-Rad, USA).

Antibacterial activity determination

Extracellular MIC: Clinical MRSA isolates (B1-1, stored in the National Veterinary Drug Residues Reference Laboratory of Huazhong Agricultural University) were recovered and passaged in LB broth medium. MRSA at a level of 2×10^5 CFU/mL was added to 96-well dishes. Different doses of double dilution of Lev, Lev-PCN-224, Lev-GaPR-PCN and HA-Lev-GaPR-PCN were added to the 96-well dish respectively. The 96-well dish was then irradiated with a 660 nm laser at a power of 100 mW/cm^2 for 10 min. After a break of 30 min, it was irradiated again for 10 min. The 96-well dish was then incubated at 37 °C for 24 h. The extracellular MIC was determined using the lowest dose that completely inhibits the proliferation of MRSA by determining the OD value at 600 nm.

Intracellular MIC: RAW 264.7 cells were inoculated at a density of approximately 10^4 cells per well from 96-well culture plates and then infected with approximately 10^5 MRSA per well. After the 2-h infection, 200 μg/mL gentamycin was added to the DMEM culture medium to completely eliminate the extracellular MRSA. Different doses of double dilutions of Lev, Lev-PCN-224, Lev-GaPR-PCN and HA-Lev-GaPR-PCN (calculated as Lev) were each incubated for 24 h to determine the intracellular MIC. PBS was used as a control. After a 4-h addition of Lev and nanosystems, all cells were irradiated twice for 10 min with a 660-nm laser at a power of 100 mW/cm^2 (30-min break between the two irradiations). After 18 h of cultivation, the extracellular MRSA were removed by washing three times with PBS, and the intracellular bacteria were determined by the OD value at 600 nm of the cell lysates. The intracellular MIC was determined by the lowest dose that completely inhibited the proliferation of intracellular MRSA by determining the OD value.

Safety study

To evaluate the safety of mice, healthy Balb/C mice were injected with HA-Lev-GaPR-PCN via a tail vein for 14 d at a daily dose of 36 mg/kg (calculated as Lev). At the end of the experiment, the hematological and biochemical parameters were analyzed. Heart, liver, spleen, lung and kidney were weighed after necropsy to obtain organ coefficients. The heart, liver, spleen, lung and kidney were then removed, fixed with 4 % formaldehyde and

dehydrated with ethanol. After kerosene embedding, the tissues were sliced for subsequent staining and analysis.

Result

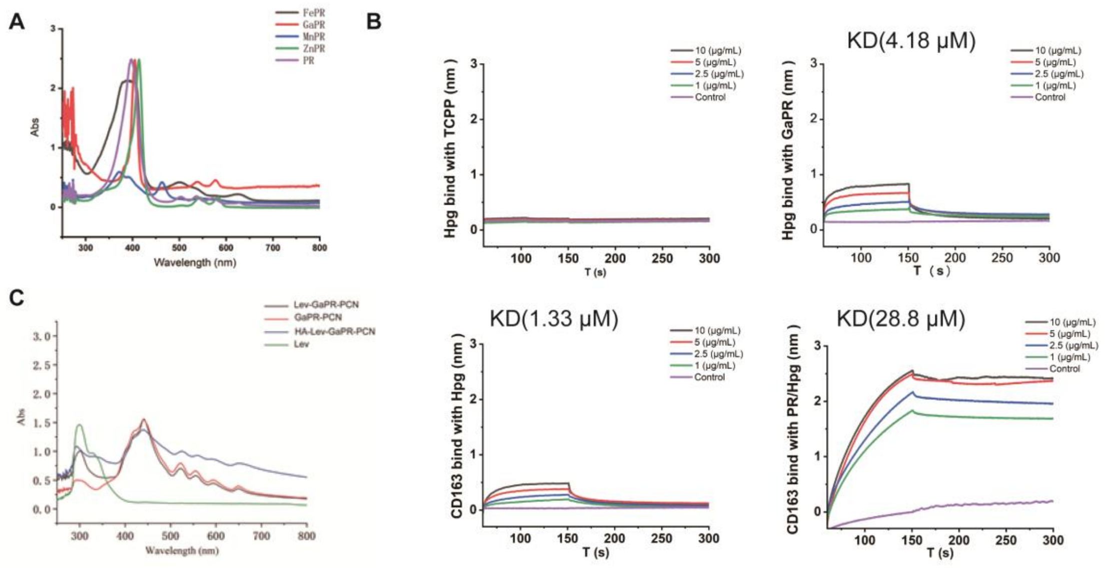

Figure S1. Design and screening of targeting nutritional competitive antibiotics (trojan horse). A) UV-vis DRS spectra of FePR, GaPR, MnPR, ZnPR, and protoporphyrin. **B)** The binding efficiency of of TCPP and GaPR with Hpg and CD163 determined by BLI. **C)** UV-vis DRS spectra of Lev, GaPR-PCN, Lev-GaPR-PCN, and HA-Lev-GaPR-PCN.

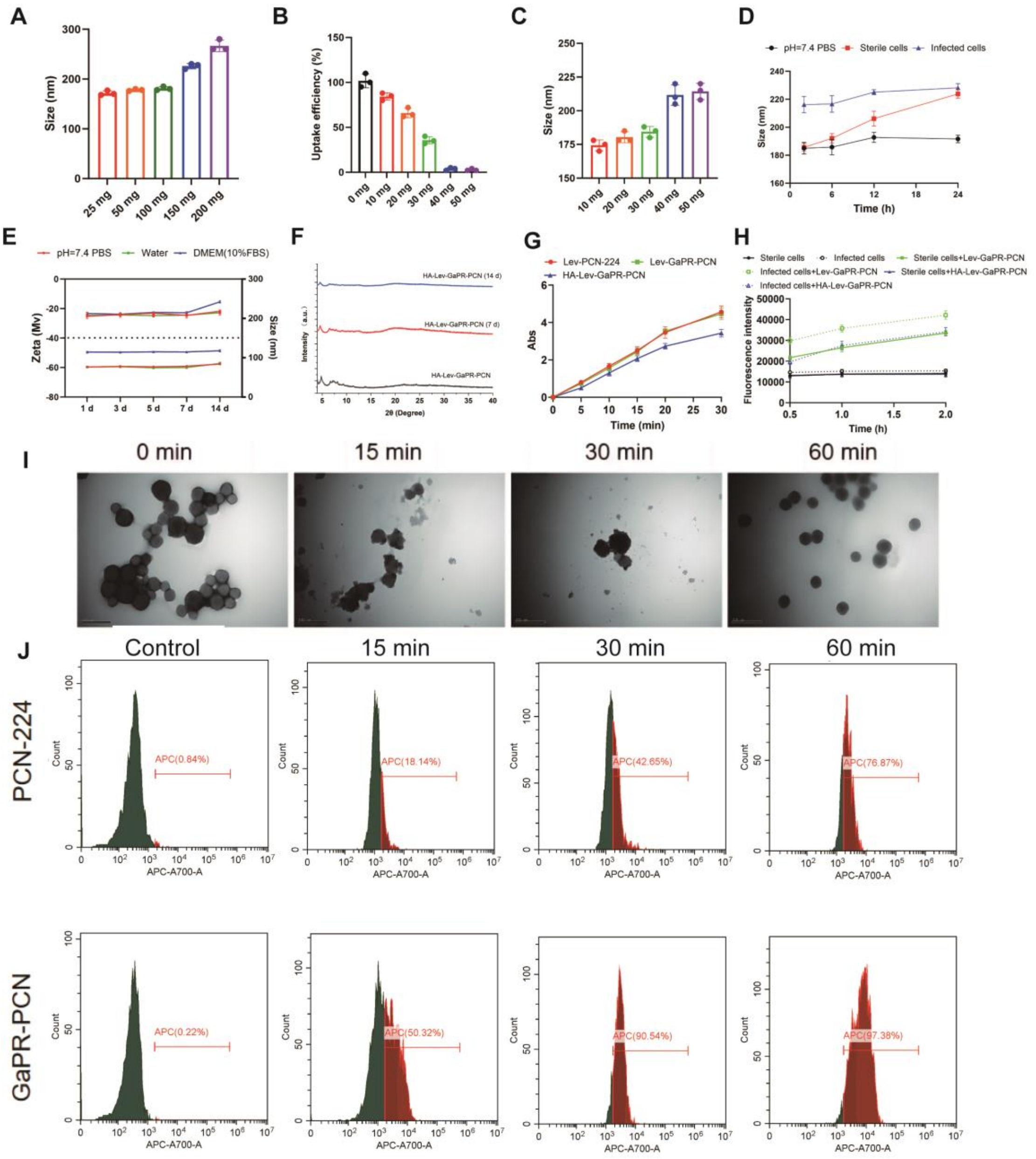

Figure S2. Characterization and programmed release of different nanosystems. A) DLS size of GaPR-PCN added 25-200 mg GAPR. **B)** Cellular uptake of various HA-Lev-GaPR-PCN by sterile RAW 264.7 cells. and HA-Lev-GaPR-PCN **C)** DLS size of HA-Lev-GaPR-PCN added 10-50 mg HA-TK-NH_2. **D)** DLS of Lev-GaPR-PCN in PBS, sterile cells, and MRSA infected cells. **E)** DLS and zeta potential of HA-Lev-GaPR-PCN in PBS, water, and DMEM (10% FBS) for 14 d. **F)** XRD pattern of HA-Lev-GaPR-PCN in

DMEM (10% FBS) for 14 d. (graph changed to arbitrary unit). **G)** Absorbance value of singlet oxygen produced by Lev-PCN-224, Lev-GaPR-PCN and HA-Lev-GaPR-PCN incubating with pH7.4 PBS. **H)** Fluorescence intensity of intracellular singlet oxygen when Lev-PCN-224, Lev-GaPR-PCN and HA-Lev-GaPR-PCN incubating with sterile and infected RAW 264.7 cells. **I)** TEM images of of HA-Lev-GaPR-PCN after incubating with MRSA infected cells for different time points. **J)** Flowcytometry images of APC-A700 fluorescence counts on MRSA after co-incubating with PCN-224 and GaPR-PCN for 15 min, 30 min, and 60 min.

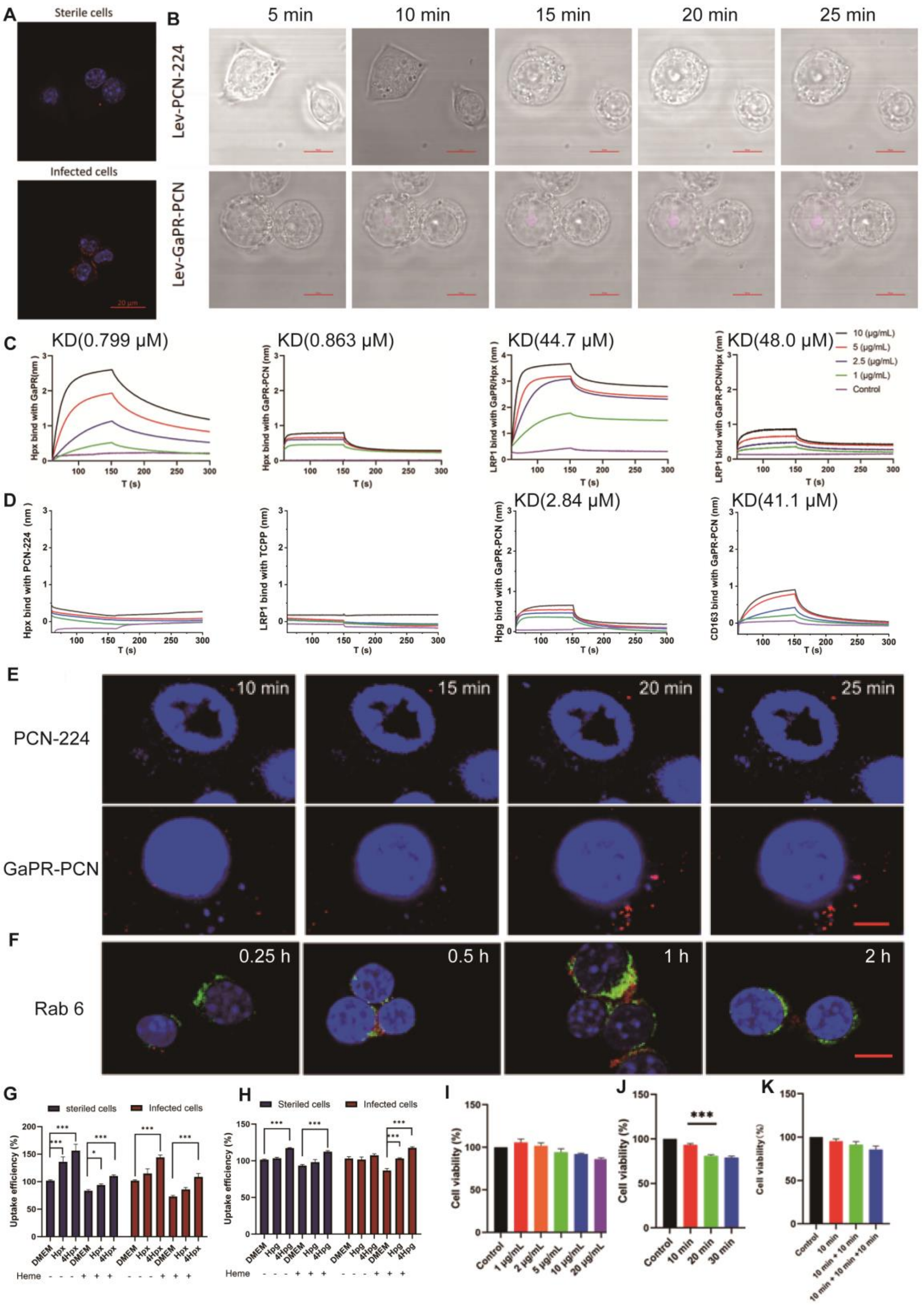

Figure S3. Targeting performance and mechanism of nanosystems to infected cells and intracellular MRSA. A) CLSM images of HA-Lev-GaPR-PCN inside RAW 264.7 after incubating for 4h. **B)** Continuous

confocal fluorescence microscope images for transporting of PCN-224 and GaPR-PCN into sterile RAW 264.7 for 5-25min. Scale bars, 10 μm. **C)** The binding efficiency of GaPR and GaPR-PCN with Hpx, LRP1, as well as their combined complexes with LRP1 determined by BLI sensorgrams. **D)** The binding efficiency of TCPP, GaPR and GaPR-PCN with Hpx, LRP1, and Hpg, as well as their combined complexes with CD163 determined by BLI sensorgrams. **E)** Continuous confocal fluorescence microscope images of HA-GaPR-PCN transport into MRSA infected RAW 264.7 cells during 125-145 min. Scale bars, 5 μm. **F)** Intracellular co-localization of GaPR-PCN with trans-Golgi network. Scale bars, 10 μm. **G-H)** Cellular uptake of Lev-GaPR-PCN by sterile and MRSA-infected RAW 264.7 cells, when Hpx, Hpg, and heme were added. Added heme (+),Do not added heme (-). **I)** RAW 264.7 viability after treatment with different concentrations of HA-Lev-GaPR-PCN (1.0, 2.0, 5.0, 10.0, and 20.0 μg/mL). **J)** RAW 264.7 viability after treatment with HA-Lev-GaPR-PCN (10 μg/mL), under irradiating at 660 nm for 10, 20, and 30 min. **K)** RAW 264.7 viability after treatment with HA-Lev-GaPR-PCN (10 μg/mL), under irradiating for 10, 10+10 (a break of 30 min), and 10+10+10 (two break of 30 min) at 660 nm.

Table S1. Primer sequences for *isd b* and *Isd h*

Gene	Forward Sequence (5'to3')	Reverse Sequence (5'to3')
isdb	*GTCATCACTAGGCGTTGCATCTG*	*GCTTCTGTATTTGTACCACCTGTTTCT*

Isdh	*CCCGGATCCGAACATCTGCCGAGTGATATTC*	*CGGGTCGACCAGTAAGATTATTTTCAATTAG*
